# The Binding of the High Affinity Radioligand Fallypride to the D2-dopamine Receptor is Sensitive to Injected Mass – a PET Study in Rodents

**DOI:** 10.1007/s11307-026-02096-7

**Published:** 2026-03-16

**Authors:** Miklós Tóth, Lenke Tari, Sangram Nag, Tian Qiu, Zhisheng Jia, Jenny Häggkvist, Jogeshwar Mukherjee, Andrea Varrone, Christer Halldin, Lars Farde

**Affiliations:** 1 Department of Clinical Neuroscience, Center for Psychiatry Research, Karolinska Institutet and Stockholm County Council, Stockholm, 171 76 Sweden; 2 Department of Radiological Sciences, Preclinical Imaging Center, University of California-Irvine, Irvine, CA USA; 3 Department of Biophysics and Radiation Biology, Semmelweis University, HUN-REN TKI, Budapest, 1094 Hungary

**Keywords:** Positron emission tomography (PET), Fallypride, D2-dopamine receptor, Mass effect, Molar activity, Mouse

## Abstract

**Purpose:**

Fallypride is a widely used high affinity radioligand for quantification of D2-dopamine receptor binding in small animal PET imaging. To examine the effect of mass both [^18^F]fallypride and [^11^C]fallypride was injected in mice over a wide range of molar activity and the binding potential (BP) was compared.

Procedures.

Eight mice (C57BL/6 J) went through three PET measurements within two weeks. [^11^C]fallypride with the highest possible molar activity (MA) and [^18^F]fallypride with normal and lower molar activity (lowerMA).

**Results:**

The binding of [^11^C]fallypride was highest with a BP_ND_ of 13.5 ± 1.1 BP_ND_. The binding of [^18^F]fallypride was lower, 8.6 ± 2.2 BP_ND_ in the normal condition and 5.3 ± 1.3 BP_ND_ at the lowerMA condition. By consequence, BP_ND_ showed a strong negative correlation with injected mass (R2 = 0.95, *P* < 0.05). Binding data were entered in a Scatchard analysis yielding a B_max_ of 62 pmol/g and a K_D_ of 0.25 nM.

**Conclusion:**

In this PET study in mice the average radioligand occupancy was estimated to 19% even for ^11^C-labeled fallypride despite the high MA of 153.3 GBq/µmol and low injected mass of 0.03 µg. Therefore, high affinity radioligands should be applied with care in small animal PET studies and radiochemistry has to be at its best to assure that tracer conditions are met in small animal imaging PET imaging.

## Introduction

Radiolabeled tracers have long been used for molecular brain imaging by positron emission tomography (PET) in humans. During the past two decades an increasing number of studies have been conducted by using dedicated PET-systems in small animals [1–3].

When comparing small animal and human PET imaging one obvious difference is the size. For instance, the body weight of a rat is about 300 times lower, and the brain weight is about 700 times lower than in humans. To allow for detailed small animal imaging the PET manufacturers have developed tailored detector designs allowing for higher sensitivity and improved resolution. In addition, the applied reconstruction matrix includes a smaller voxel size i.e. about 0.06 mm^3^ in small animal imaging vs 2 mm^3^ or larger in human imaging. However, to achieve the same reliability the same number of coincidences should ideally be detected from a typical rodent “voxel” as in human studies [1]. By consequence the injected radioactivity (Mbq/kg) and radioligand mass (ug/kg) is routinely higher in small animal imaging.

The mass injected in a human subject may not deem an issue, since the radioligand usually occupies only a few percent of the targeted protein (a “tracer dose”). However, if more radioactivity is injected, such as in small animal PET imaging, the radioligand occupancy will be higher and approach the non-linear part of the saturation curve. The measured signal will consequently be sensitive to the mass injected i.e. there will be a “mass effect” dependent on the molar activity of the synthesized product [1]. For instance, for the study of the striatal D2-dopamine receptor in mice it has been suggested that there has to be a tenfold increase in the molar activity of the reference radioligand [^11^C]raclopride when compared to typical human PET experiments [2].

An additional challenge is the need for imaging of regions with a low density of the target protein. Whereas a radioligand like [^11^C]raclopride is useful for measuring the high density of the D2-dopamine receptor in the striatum, radioligands with higher affinity are required for the low density extrastriatal regions [4, 5]. For this purpose, radioligands such as [^11^C]FLB457 and [^18^F]fallypride have been developed [6–8].

The binding of [^18^F]fallypride to the D2-dopamine receptor has been quantified in applied studies in rodents, nonhuman primates and humans [9, 10]. However, small animal imaging with [^18^F]fallypride has not been critically examined with regard to a potential “mass effect” [4, 11–15].

We have recently set up the radiosynthesis of [^11^C]fallypride which can be radiolabeled to a higher molar activity than [^18^F]fallypride. This combination makes it possible to examine the binding of radiolabeled Fallypride over a wide range of molar activity.

In this study our objective was to examine the effect of mass injected on fallypride binding. In each of eight mice, the binding potential (BP) after [^11^C]fallypride injection as well as after two injections of [^18^F]fallypride (early and late after synthesis) was compared.

## Materials and Methods

### Animals and Design

Animal experiments were designed and conducted according to the Swedish National Board of Laboratory guidelines and approved by the Ethics Review Board of Northern Stockholm, Sweden (N172/14). Eight 3 months old C57BL/6 J mice were included. The animals were housed in a group, in a thermoregulated (~ 22 °C) and humidity-controlled environment, under a 12 h/12 h light/dark cycle with access to food and water ad libitum, within individually ventilated cages.

Each mouse was examined by PET on three experimental days within two weeks and with at least 2 days in between experimental days. On two days [^18^F]fallypride was injected either immediately after production or after 2 h, i.e. about one half-life of F18. The order of [^18^F]fallypride experimental days was random and the experimental day with [^11^C]fallypride was the last in each animal. The body weight was 23.66 ± 0.71 g, 23.99 ± 0.87 g and 23.98 ± 0.94 g at the first, second and third PET measurements, respectively.

### Radiochemistry

The precursor 5-(3-fluoropropyl)−2-hydroxy-3-methoxy-N-[(2S)−1-(2-propenyl)−2-pyrrolidin yl]methyl] benzamide (desmethyl-fallypride) was synthesized at Brain Imaging Center, Department of Psychiatry and Human Behavior, University of California-Irvine, Irvine, CA 92697, USA, according to a previously described method(*16*).

The precursor (S)-N-[(l-Allyl-2-pyrrolidinyl)methyl]-S-(3-toluene sulfonvloxypropyl)−2,3-dimethoxybenzamide (tosyl-fallypride) and the non-radioactive reference standard (S)-N-[(1-allyl-2-pyrrolidinyl)methyl]−5-(3-fluoropropyl)−2,3-dimethoxybenzamide (fallypride) was purchased from PharmaSynth AB, Estonia. Liquid chromatographic analysis (LC) was conducted utilizing a Merck-Hitachi gradient pump and a Merck-Hitachi L-4000 variable wavelength UV detector. All other chemicals and reagents used in the analysis were obtained from commercial sources and were used without any additional purification.

## Synthesis of [^11^C]fallypride

[^11^C]Fallypride was synthesized according to a previously published method with small modifications [17]. In short, [^11^C]CH_3_OTff at room temperature was trapped in the reaction vessel contained the appropriate desmethyl fallypride precursor (0.5–0.7 mg) and 0.5 M NaOH (6 µL) in acetone (400 µL). The mixture was then heated at 50 °C for 120 s. After synthesis, the residue was diluted with sterile water (2 mL) and injected into the HPLC injection loop for purification. The desired product [^11^C]fallypride, was collected in a bottle containing sterile water (50 mL). The collected mixture was further purified by passing it through a SepPak solid-phase extraction (SPE) cartridge and eluted with 1 mL of ethanol into a sterile vial containing sterile saline (9 mL). The formulated product was then sterile filtered using a Millipore Millex® GV filter unit (0.22 μm) for subsequent use. The radiochemical yield was more than 500 MBq of the final product with purity > 95%.

## Synthesis of [^18^F]fallypride

Fluorine-18 fluoride ([^18^F]F-) was generated by following a previously published method [18]. To the residue or [^18^F]F^−^, the corresponding precursor (2.0 mg, 0.01 mmol) in DMSO (600 µL) was added to the closed reaction vessel followed by heating at 130 °C for 20 min. Afterward, the reaction vessel was cooled to room temperature and diluted with sterile water (3 mL) before injecting the mixture into the HPLC for purification. The desired product [^18^F]fallypride, was further purified by passing through a SepPak solid-phase extraction (SPE) cartridge and was eluted with 1 mL of ethanol into a sterile vial containing sterile saline (9 mL). The formulated product was then sterile filtered using a Millipore Millex® GV filter unit (0.22 μm) for subsequent use. The incorporation yield of the fluorination reaction was more than 50% resulted > 5 GBq of the final product. The radiochemical purity was higher than 99%.

The molar activity values at time of injection are given in Table 1. In one production of [^11^C]fallypride the molar activity could not be calculated due to loss of data for two PET measurements. Therefore, only six animals are included in the analysis of [^11^C]fallypride binding.

**Table 1:** Basic experimental data listing weight, injected radioactivity (Injected RA), molar activity and injected mass

	[^11^C]Fallypride (*n* = 6)	[^18^F]Fallypride (*n* = 8)	[^18^F]F.LowerMA (*n* = 8)
Weight (g)	24.0 ± 0.9	23.8 ± 0.9	23.8 ± 0.7
Injected RA (MBq)	11.1 ± 1.1	13.0 ± 0.9	12.0 ± 0.6
Molar activity @ injection (GBq/µmol)	153.3 ± 81.7	44.9 ± 11.1	21.2 ± 4.8
Injected Mass (µg)	0.03 ± 0.02	0.11 ± 0.03	0.21 ± 0.05

### MRI and PET Imaging Procedure

The animals were anesthetized individually by inhalation of isoflurane (4–5% isoflurane in 100% oxygen) which was maintained at 1.5–2% concentration (50/50 air/oxygen) afterwards. The animals were positioned in a dedicated mouse bed that was heated through circulating warm air to maintain optimal body temperature under anaesthesia. Vital signs were monitored continuously for each animal during the experiments. The weight was measured at each experimental day (Table 1).

Mice were cannulated through the lateral tail vein and positioned in the PET system by placing the head in the center of the field of view of the detector-ring. The radioligand was intravenously injected as a bolus at start of PET data acquisition, and the cannula was immediately flushed by 0.02 mL saline. At the end of each imaging session, the animal was monitored until fully awake and returned to its cage.

### Imaging System and Reconstruction

The PET measurements were conducted using the Mediso (Mediso Medical Imaging Systems Ltd. Budapest, Hungary) nanoScan® PET/MRI and the nanoScan® PET/CT small animal imaging systems [19, 20]. Two animals were examined at the same time in the identical PET modules of the two cross-calibrated imaging systems. The acquired list mode data were reconstructed into 25 time frames (93 min scan = 4 × 10 s, 4 × 20 s, 4 × 60 s, 7 × 180 s, 11 × 360 s). The image reconstruction was made with a fully 3-dimensional penalized maximum-likelihood algorithm (MLEM; Tera-Tomo; Mediso Ltd.) with 20 iterations (without scatter or attenuation correction).

### PET and MR Data Processing and Analysis

Each animal’s dynamic PET image was co-registered to the mouse MRI template available in PMOD (PMOD Technologies Ltd., Zurich version: 3.7). Decay corrected time activity curves (TAC) were generated using the volume of interest (VOI) template in PMOD. To normalize for injected radioactivity and body weight the regional radioactivity values were expressed as standard uptake values (SUV = regional activity * body weight/injected radioactivity). The VOI for the cerebellum was defined to exclude areas where bone uptake of F18 could influence the radioactivity concentration in brain.

The outcome parameter was the non-displaceable binding potential (BP_ND_), calculated by using Logan reference model [21] with the cerebellum as reference region. Statistical analysis of BP_ND_ values was performed by using the one-way ANOVA Bonferroni’s multiple comparison post-test.

### Scatchard Analysis

Scatchard analysis was performed as described by Farde et al. (1989) [22]. In short, the total radioligand concentration in the cerebellum was used as an estimate for free radioligand concentration in the striatum (F). Specific binding (B) was defined as radioactivity in the striatum reduced with F. The values for B and F were used in a Scatchard analysis where the ratio B/F was plotted vs. F for each experimental point and followed by a linear fit. D2-dopamine receptor density (Bmax) was defined by the intercept with the X-axis and affinity (Kd) by the slope of the fitted line.

## Results

**Fig. 1:**
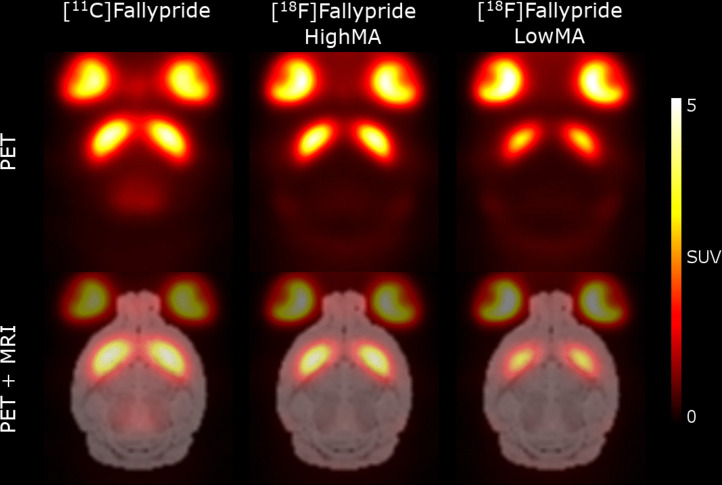
Average images of fallypride uptake at three conditions shown in horizontal sections (top) and PET overlaid on the MRI template (bottom). The number of animals included in each condition [^11^C]fallypride *n* = 6, [^18^F]fallypride *n* = 8 and [^18^F]fallypride lowerMA *n* = 8

Average images obtained in mice after injection of [^11^C]fallypride, [^18^F]fallypride and [^18^F]fallypride lowerMA are shown in Fig. 1 and overlaid on the MRI template. There was a conspicuous uptake within the eye socket as well as in the striatum. Extrastriatal dopamine D2 binding could be visually observed in the midbrain after injection of [^11^C]fallypride whereas a slight bone uptake is seen on the [^18^F]fallypride images. The visual impression of the images is that there was a slight decrease of ligand binding dependent on injected mass.

**Fig. 2:**
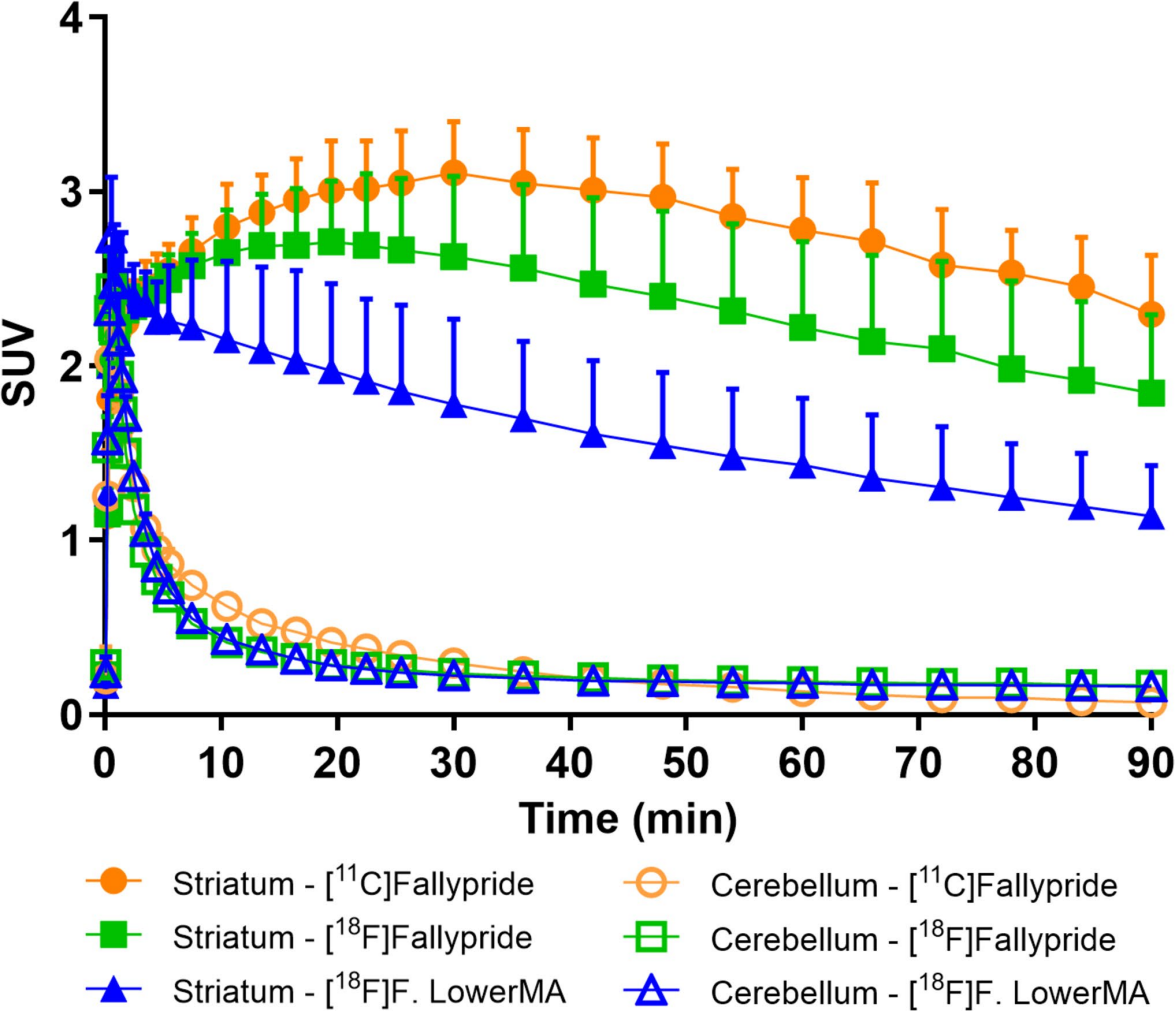
Average time radioactivity curves for striatum and cerebellum at three experimental conditions (values expressed as mean + SD). The number of animals included in each condition [^11^C]fallypride *n* = 6, [^18^F]fallypride *n* = 8 and [^18^F]fallypride lowerMA *n* = 8

Average TACs for the striatum and cerebellum were generated after injection of [^11^C]fallypride, [^18^F]fallypride and [^18^F]fallypride lowerMA (Fig. 2). The striatal radioactivity concentration was markedly reduced with increasing injected mass whereas the concentration in the cerebellum remained at a low level, or increased slightly by the end of the measurements with [^18^F]fallypride.

**Fig. 3:**
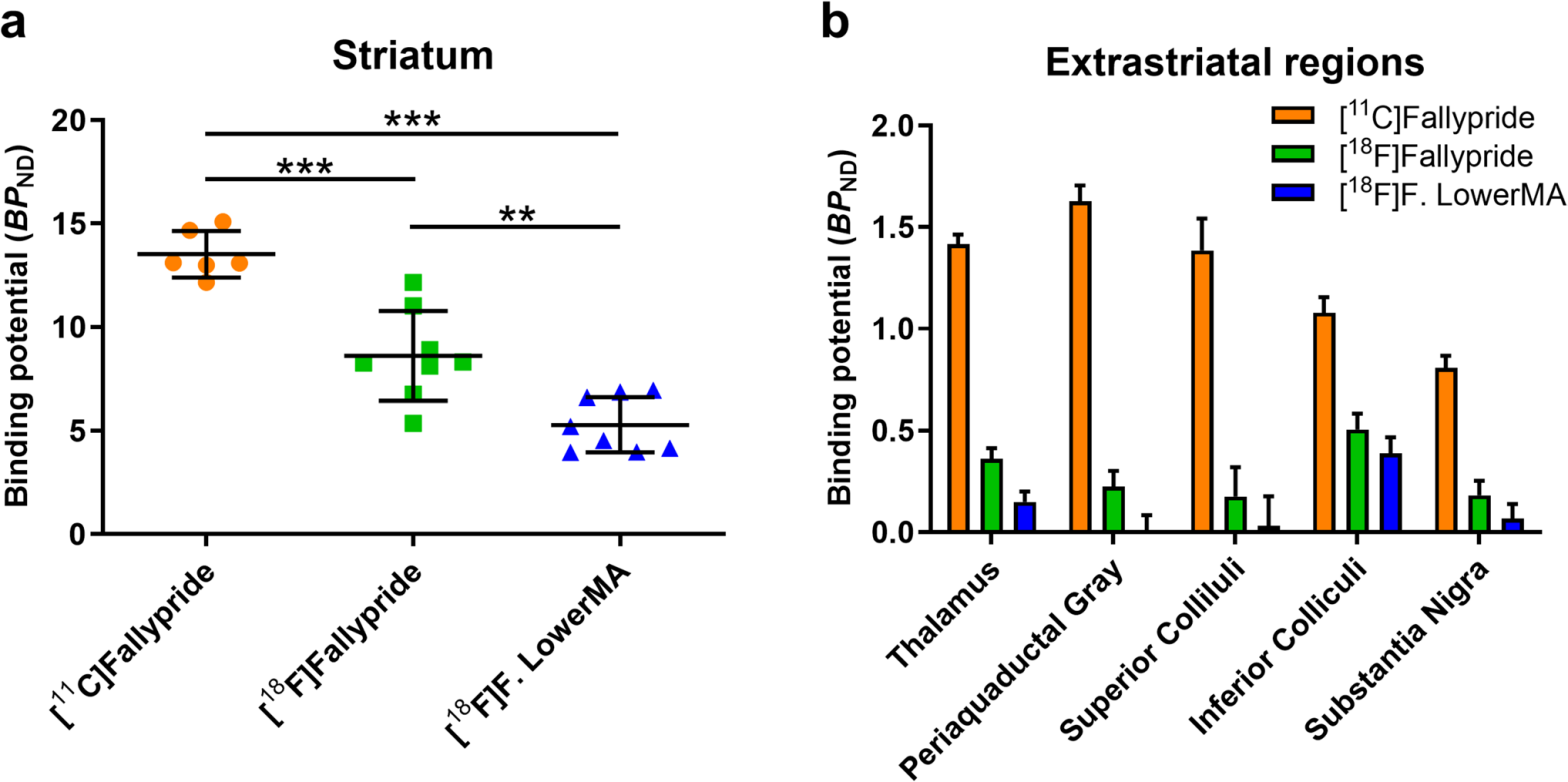
**a**. Individual BP_ND_ values calculated with Logan reference method for the striatum (** *p* < 0.05, *** *p* < 0.005) **b**. Average BP_ND_ values for extrastriatal regions. The number of animals included in each condition [^11^C]fallypride *n* = 6, [^18^F]fallypride *n* = 8 and [^18^F]fallypride lowerMA *n* = 8

The striatal binding potential (BP_ND_) values (average ± SD) were calculated by using the Logan reference method. (Fig. 3a). The BP_ND_ for the striatum was 13.5 ± 1.1 for [^11^C]fallypride (*n* = 6), 8.6 ± 2.2 BP_ND_ for [^18^F]fallypride (*n* = 8) and 5.3 ± 1.3 BP_ND_ for [^18^F]fallypride lowerMA (*n* = 8). The average injected mass corresponding to these BP_ND_ values are given in Table 1. The BP_ND_ values in extrastriatal regions were much lower and plotted in a bar graph (Fig. 3b).

**Fig. 4:**
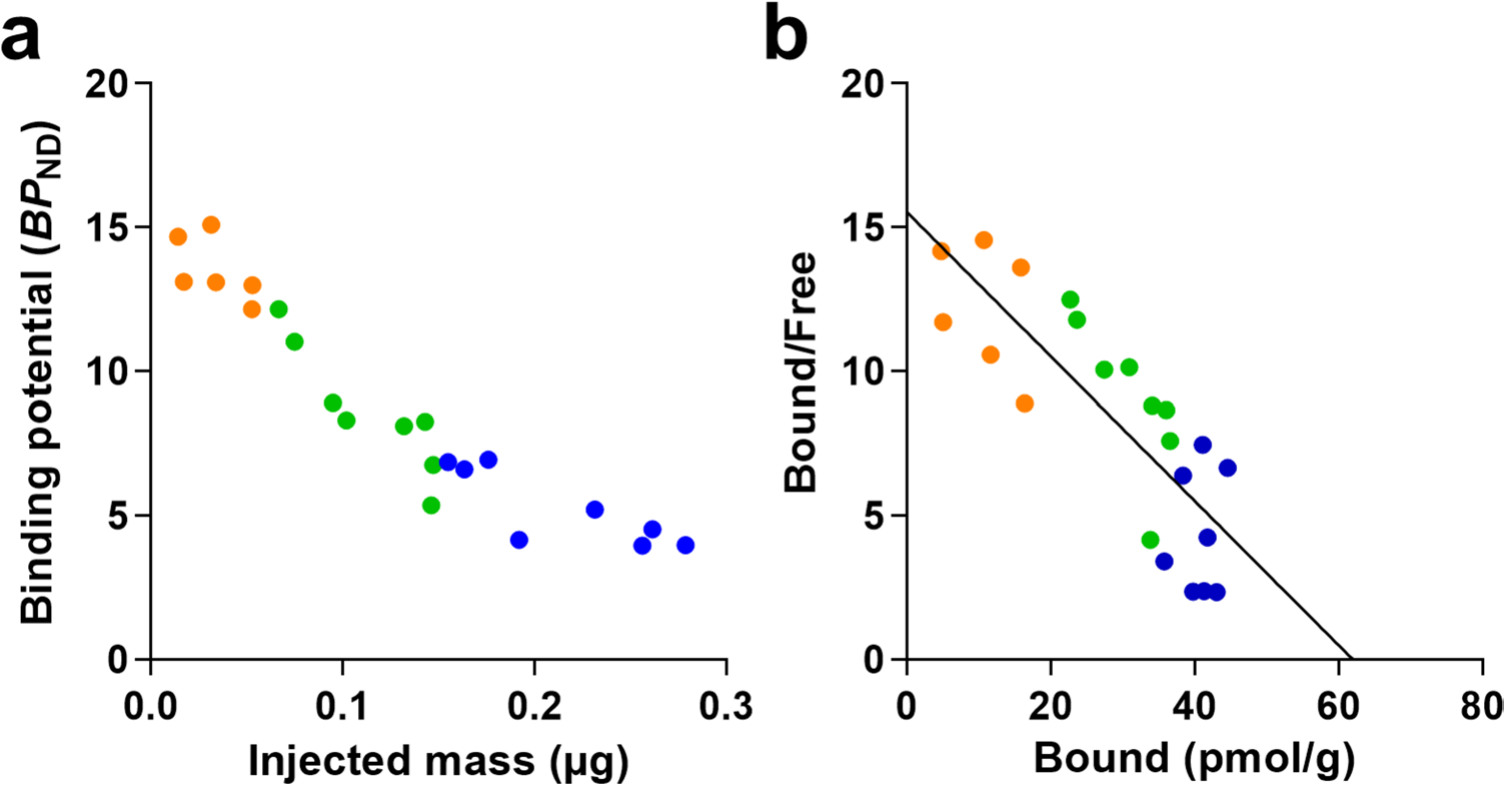
** a**. Binding potential values plotted vs injected mass for the total of 22 PET-measurements **b**. Scatchard analysis of the same data set. The dots for [^11^C]fallypride are in yellow,for [^18^F]fallypride in green and for [^18^F]fallypride lowerMA in blue)

The striatal BP_ND_ for fallypride was dependent on the mass injected as shown in Fig. 4a. A Scatchard plot based on the 22 PET-measurements is shown in Fig. 4b. D2-dopamin receptor density (Bmax) was estimated to 62 pmol/g as defined by the intercept with the x-axis and the affinity (K_D_) was 0.25 nM as defined by the slope.

## Discussion

[^18^F]Fallypride is an established radioligand for human and small animal imaging of striatal as well as extrastriatal D2-dopamine receptor binding in brain. In the present study we developed [^11^C]fallypride which can be radiolabelled to a higher molar activity than [^18^F]fallypride. The unique combination of [^11^C]fallypride and [^18^F]fallypride allowed for examination of binding over a wide range of molar activity and mass injected. It is important to point out that between the body of work done in these experiments and its publication, an advancement has been made in the field of [^18^F]Fallypride production by Huhtala et al. to achieve much higher MA levels than before. This would have negated the need to include the C11 labelled version in this study [23], however we feel that this does not take away from the conclusions of this paper.

In our experiments in mice, we found that the striatal BP_ND_ decreased markedly when going from the high MA condition with [^11^C]fallypride to lower MA condition with [^18^F]fallypride. The most likely explanation is that the mass of injected [^18^F]fallypride is sufficient to approach saturation of the D2 dopamine receptor. The BP_ND_ calculated with Logan reference method dropped from 13.5 in the [^11^C]fallypride experiments to 5.3 BP_ND_ with [^18^F]fallypride at the lower MA condition. This reduction corresponds to at least 50% radioligand occupancy at the receptor.

The concept “tracer dose” has been suggested when the occupancy of the injected ligand is below 5%. At such occupancy, variations in the radiochemistry production quality have limited impact on BP_ND_ [1, 2, 24, 25]. However, at the demonstrated high radioligand occupancy of [^18^F]fallypride the BP_ND_ will be sensitive to the production quality which can be viewed as a confounder in applied studies.

A critical condition in our study is if the injected radioactivity and molar activity is representative of applied studies in literature. While the range of injected radioactivity in our study is in line with what has been applied by other centers [15, 16, 23, 26, 27], the actual injected mass or molar activity at injection is rarely reported in literature.

Worth noting is that the hereby discussed issue of injected mass effect is not a problem when using PET radiopharmaceuticals that explore non-saturable systems. like [^18^F]Fluoride for bone surface uptake imaging. Another example for which injected mass is a limited problem is the study of intermediate saturable systems such as with [^18^F]Fluoro-2-deoxyglucose (FDG) that measure changes in glucose consumption [1, 2].

A slight difference in radioligand washout from the cerebellum might still be observable at the end of data collection, when C11 and F18 curves are compared. This is most likely due to spillover from bone uptake and may have had a negligible effect on BP_ND_ estimation.

An interesting observation was that binding in extrastriatal regions was conspicuous after injection of [^11^C]fallypride having the highest molar activity concentration, and a significantly lower amount of injected mass. BP_ND_ measurements in low receptor density areas should thus be possible as well. This potential is less evident for [^18^F]fallypride.

An attempt was made to use Scatchard analysis to quantify fallypride binding to the D2-dopamine receptor in the striatum. Bmax was estimated to 62 pmol/g which is somewhat higher than the Bmax estimates of about 30 pmol/g seen in humans [28, 29] and 34 pmol/g in mice using [^3^H]spiperone* in vitro* [30]. It has been suggested that for fallypride binding in brain there may be a D3 component of around 20%, that could be subject to species differences [27].

Using data obtained in the Scatchard plot analysis (Fig. 4b) we estimate that the mass related occupancy in our experiments with [^18^F]fallypride was quite high: 41% and 64% at average, [^18^F]fallypride vs [^18^F]fallypride lower MA respectively. Importantly even with [^11^C]fallypride the average occupancy was 19% despite a molar activity of 153.3 GBq/µmol and an injected mass of 0.03 µg (Table 1).

There is a possibility to decrease injected mass by lowering the radioactivity injected. However, such approach might reduce image quality. Ideally, MA must be further improved to assure the tracer conditions required for small animal imaging.

## Conclusion

Using [^11^C]fallypride and [^18^F]fallypride at different molar activity conditions we found that [^18^F]fallypride binding to the D2-dopamine receptor is sensitive to injected mass in mice. Our results highlight the need to control the molar activity levels with care to ensure consistency between injections in applied small animal imaging studies.

## Data Availability

The datasets generated during and/or analyzed during the current study are available from the corresponding author on reasonable request.
